# Modification of a Low-Cost Pipetting Robot for Nanoliter Liquid Handling and Autosampling for Liquid Chromatography-Mass Spectrometry

**DOI:** 10.1002/jssc.70379

**Published:** 2026-02

**Authors:** Nathaniel B. Axtell, Kei G. I. Webber, Thy Truong, Hsien-Jung L. Lin, Andrew Sandberg, Spencer Martin, Xiaofeng Xie, Siqi Huang, Chao Wang, Ryan T. Kelly

**Affiliations:** 1 Department of Chemistry and Biochemistry, Brigham Young University, Provo, Utah, USA; 2 MicrOmics Technologies, LLC, Spanish Fork, Utah, USA

**Keywords:** autosampler, lab automation, LC-MS, nano-LC, single-cell proteomics

## Abstract

Automated liquid handling can achieve higher precision for low-volume dispensing than hand-held pipetting while increasing sample processing throughput and avoiding human error. Current commercial liquid-handling platforms range in cost from tens to hundreds of thousands of dollars, which can be prohibitive for smaller research groups. Home-built systems offer customized functionality but require significant technical knowledge and specialized parts. We reduced the burden of developing homebuilt systems by adapting the hardware and software of a low-cost open-source platform, the Opentrons OT-2. The modifications extend accurate pipetting for the OT-2 to the low nanoliter range, enabling sensitive sample preparation. We then augmented the capabilities of this platform by incorporating control of two-position valves and selector valves for autosampling applications. Our modified system is flexible and can be readily configured to meet the needs of novel LC applications. We demonstrated the system for both sub-microliter sample preparation and autosampling for nano-LC-MS applications. The software developed to control the modified system can be adapted to other custom liquid handling and separations platforms.

## 1 | Introduction

The growing interest in single-cell and spatial proteomics, as well as other sample-limited applications [1–5], necessitates the development of strategies that prioritize high sensitivity. Recent advances in separations [6–15], MS instrumentation [16–20], and data acquisition [17, 18, 21] have improved analytical sensitivity, but these gains depend on adequate sample recovery from sample preparation and complete injection into the LC system [22, 23]. Traditional proteomic sample preparation strategies employ steps that are optimized for volumes typical of bulk proteomic workflows, such as using spin columns for sample cleanup [24]. These strategies incur analyte losses that are inconsequential when working with abundant samples, but which become significant when performing trace proteomic analyses. To reduce sample losses caused by surface adsorption, we developed the nanoPOTS workflow [25], in which samples were prepared without transfer steps in sub-microliter volumes, with commercial well plates being replaced by specialized glass chips. Another strategy to avoid losses is to use carrier proteins that can absorb the losses [26–28]. Leduc et al. later introduced nPOP [29], which combines the principles of nanoPOTS with tandem mass tag labeling on unpatterned hydrophobically treated glass slides for increased throughput. These one-pot, low-volume methods in single-cell proteomics (SCP) experiments have benefitted from the development of commercial platforms such as the CellenOne [30] and the Tecan Uno [31] and Duo systems, which enable precise handling of sub-microliter and even sub-nanoliter volumes.

The above-mentioned developments have enabled the rapid growth of the SCP field. Unfortunately, commercial liquid handling systems commonly used in SCP workflows can be prohibitively expensive for labs with limited resources [32, 33], often costing hundreds of thousands of dollars. Additionally, such systems are typically designed to meet the needs of existing applications, with hardware or software limits that may prohibit the customization required for novel approaches [34]. For example, introducing samples into commercial LC systems requires the samples to either be prepared in or transferred to standardized well plates, which are not optimal for all SCP strategies [35]. This challenge can be somewhat mitigated by careful selection of well plates with low protein-binding properties [36–39]. Alternatively, home-built systems, such as the nanoPOTS autosampler, can achieve liquid handling performance comparable to commercial systems and can be designed to meet the needs of unique workflows, such as sampling directly from custom nanowell slides [40]. However, developing such systems can be time intensive and requires significant technical expertise.

To enable custom workflows without requiring development of robotic systems from the ground up, we have experimented with modifying low-cost commercially available liquid handlers. Previously, we reported our work to modify a pipetting robot (Opentrons OT-1) for low-nanoliter liquid handling using only a glass syringe and 3D-printed parts [41]. Here, we have extended this work by adapting an upgraded version of the platform, the OT-2, for both nanoliter liquid handling and low-volume autosampling for nanoflow liquid chromatography (nanoLC) applications. The OT-2 has an established record of being adapted for novel approaches, such as DNA-BOT [42], AssemblyTron [43], and others [44, 45].

Unlike the OT-1, newer Opentrons systems use electronic pipettes with a combined range of 1–1000 μL [46]. This work began by modifying an OT-2 pipette to instead act as a syringe pump, similar to our previous work, effectively extending the liquid handling capabilities to the sub-microliter range. Because OT-2 pipettes are interchangeable by design, this approach preserved the native operation of the system, a notable improvement over our OT-1 adaptation. We characterized the sub-microliter liquid handling capabilities of the modified pipette using fluorescencebased assays, achieving reproducible results for aliquots as small as 20 nL. Next, we incorporated actuators with nanovolume valves into the system to enable autosampling for nano-LC-MS. We estimate the total cost of our system to be below $20K, based on current market pricing. Finally, we demonstrated the system’s applicability to diverse workflows by injecting aliquots of human protein digest using both overfilling and underfilling of the sample loop, thus demonstrating the autosampling capabilities for both excess or volume-limited samples. We injected protein lysate samples ranging from 250 pg to 10 ng, containing protein amounts in the range of single cells to tens of cells. Together, this work promotes the feasibility of low-cost alternatives to automate low-input proteomic workflows.

## 2 | Materials and Methods

### 2.1 | Chemicals and Materials

Sodium fluorescein was purchased from Sigma-Aldrich (St. Louis, MO). Pierce HeLa Protein Digest Standard and sodium borate were purchased from Thermo Fisher (Waltham, MA). LC–MS grade water with 0.1% formic acid (solvent A) and acetonitrile with 0.1% formic acid (solvent B) were purchased from Honeywell (Charlotte, NC).

Gastight 25 μL 1702 syringes were purchased from Hamilton (Reno, NV). Fused silica capillaries were purchased from Molex (Lisle, IL). The eight-port selector valve, the six-port, two-position valve, and both valve actuators were from VICI (Houston, TX). Clear-bottomed 384-well plates were purchased from Corning (Part No. 3544, Corning, NY). LoBind 384-well PCR plates and adhesive cover foils were from Eppendorf (Part No. 0030129547, Hamburg, Germany). MicroTight Unions were from IDEX (Northbrook, IL).

### 2.2 | Autosampler Configuration

**FIGURE 1 |:**
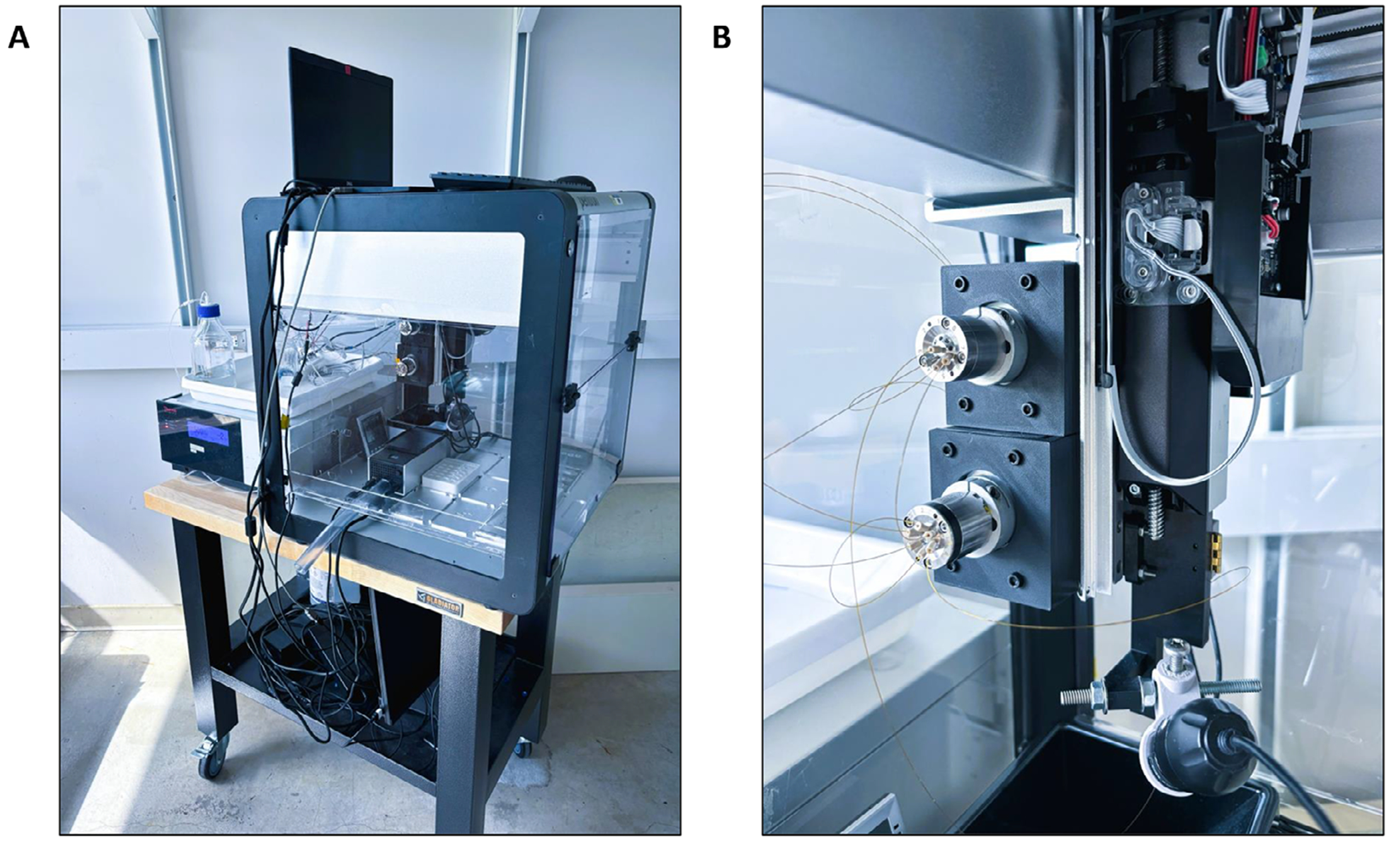
Modification of an Opentrons OT-2 system for liquid handling and autosampling of sub-microliter volumes. (A) A transfer line was run from an HPLC pump into the enclosure of a modified OT-2. (B) Valve actuators were mounted to the OT-2’s Z axis frame to enable nanoLC autosampling.

**FIGURE 2 |:**
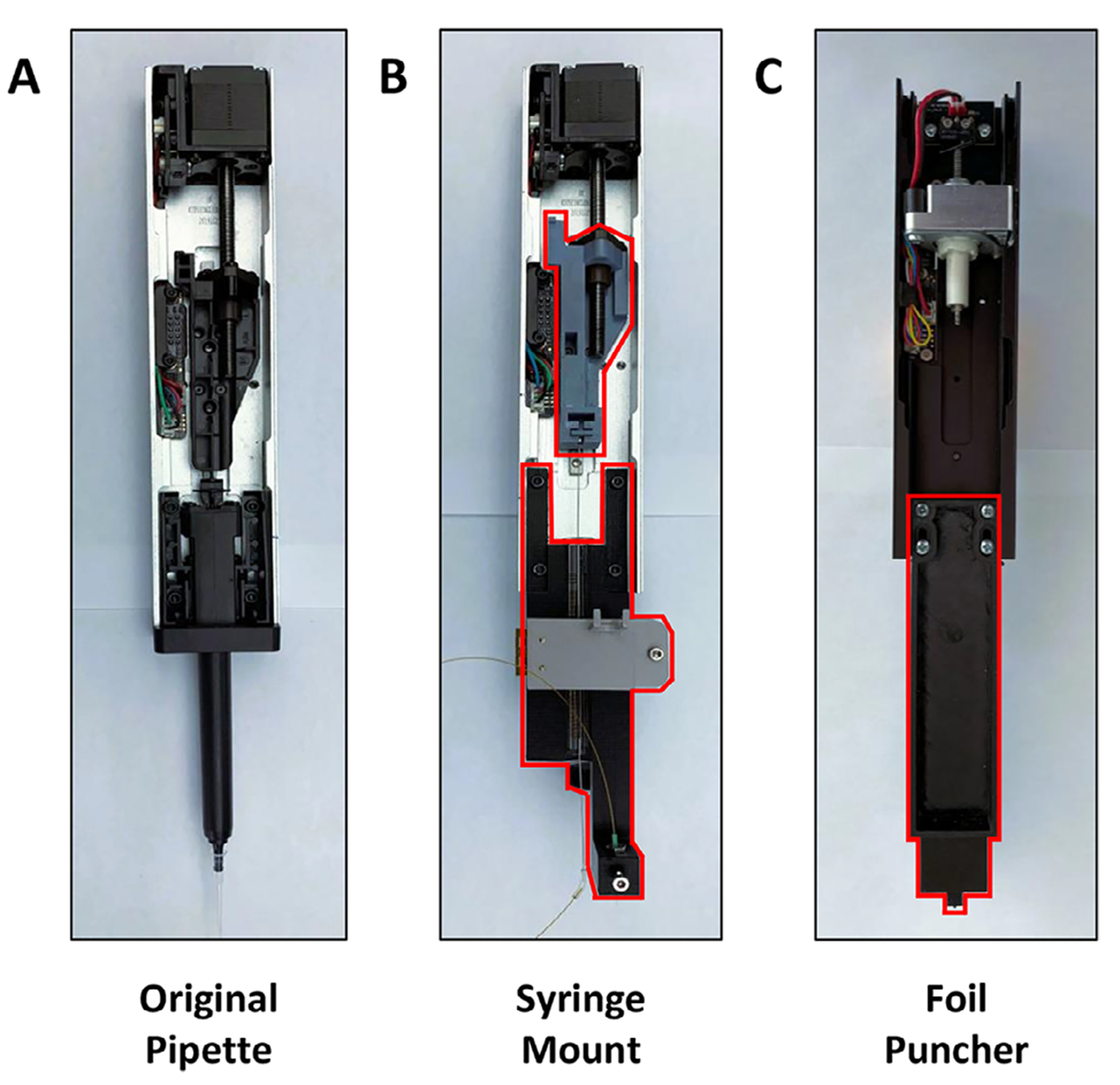
Adapting Opentrons OT-2 electronic pipettes for sub-microliter liquid handling. Parts of (A) an Opentrons Gen2 electronic pipette were replaced by (B) custom 3D-printed parts (syringe mount and plunger actuator) to operate as a syringe pump, enabling reproducible handling of volumes as low as 20 nL. (C) A Gen1 pipette was similarly modified to function as a foil puncher.

An Opentrons OT-2 was modified with 3D-printed parts and modular hardware (Figure 1) as follows. The plunger and barrel of an OT-2 Gen2 pipette were replaced with mountings for a 25-μL syringe (Figure 2). The syringe needle was fitted with a MicroTight Union (IDEX) to connect to fused silica tubing. For basic liquid handling operations, a 40-cm-long, 100-μm-i.d. silica sampling needle was connected directly to the syringe.

**FIGURE 3 |:**
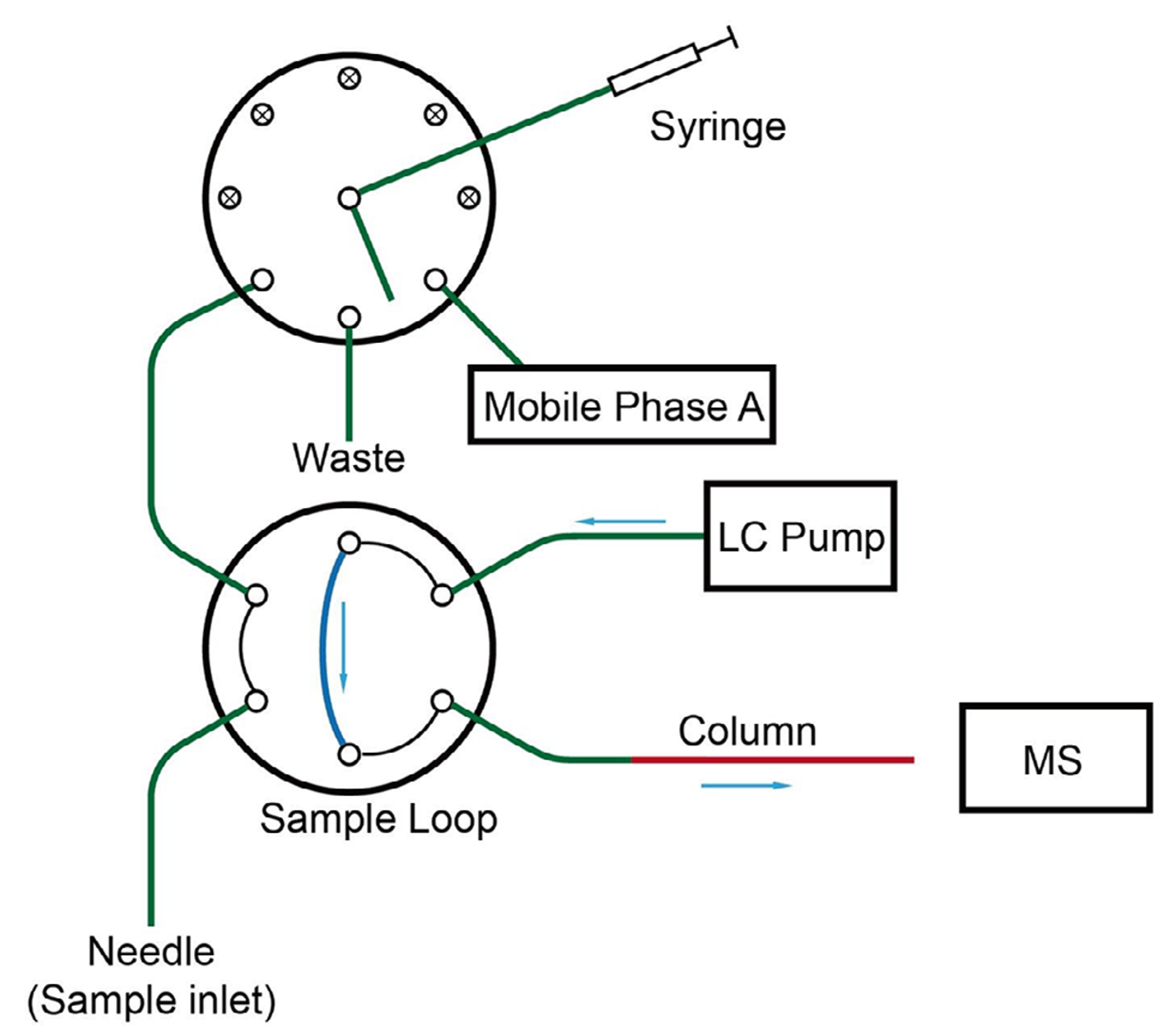
A schematic view of the valve connections for the OpenSampler system. A motor-controlled syringe aspirates samples into the sample loop. Switching the sample injection valve places the sample loop online with the pump and separation column.

For autosampling, a two-valve system was set up following the design shown in Figure 3. The syringe was connected to a selector valve by a 50-cm-long, 200-μm-i.d. fused silica transfer line. The selector valve was connected to a two-position valve via a 15-cm-long, 200-μm-i.d. transfer line. A 13-cm-long, 100-μm-i.d. fused silica capillary was connected to the two-position valve to serve as a 1-μL sample loop. The sampling needle was connected to the two-position valve.

A 75-cm-long, 20-μm-i.d. capillary transfer line was connected between the two-postion valve and a 55-cm-long, 20-μm-i.d. nanoviper line (Thermo Fisher), which was in turn plugged into the outlet of an Ultimate 3000 HPLC pump (Thermo Fisher). Another 75-cm-long, 20-μm-i.d. transfer line was connected to the two-position valve. The other end of the transfer line was connected to a 15-cm-long, 20-μm-i.d. fritted capillary, which was in turn connected to the separation column. The fused silica analytical column was 15-cm-long, 75-μm-i.d. with an integrated emitter and packed with 1.6 μm diameter C18 particles having a pore size of 100 Å (MicrOmics Technologies, LLC, Spanish Fork, Utah).

### 2.3 | Calibration

Calibration of labware was performed whenever new labware was placed in the system or if labware placements were rearranged. Well plate calibrations require all wells to be evenly spaced and consist of defining locations for three-corner wells. The system tests the calibration by moving to the fourth corner well. The user can then accept the calibration or recalibrate as needed. Calibrated labware will be listed in the main labware window. Users can save calibrations to a file for future use or recalibrate when starting the program, if preferred. Saved calibration files must be loaded into the system whenever software is restarted.

Syringe calibrations require homing the syringe motor and then defining a maximum and minimum position for the plunger as well as a preferred reference position. Syringes must be recalibrated whenever software is restarted to ensure the proper range of motion.

### 2.4 | Liquid Handling Characterization

Aliquots of sodium fluorescein (500, 100, 50, 20, 10, and 5 nL of 0.2, 1, 2, 5, 10, and 20 mM, respectively) were dispensed into wells prefilled with 20 μL of 50 mM sodium borate buffer to produce final concentrations of 5 μM in each target well. Calibration standards were prepared at concentrations of 3, 4, 5, 6, and 7 μM and hand pipetted in 20 μL aliquots with five replicates of each standard. The well plate containing the dispensed mixtures and calibration standards was cooled to 4°C throughout the preparation process using an OT-2 tempdeck module. Following preparation, the plate was placed in a PlateFuge microcentrifuge for 1 min to eliminate any bubbles. Finally, fluorescence measurements were measured with a Synergy Neo 2 plate reader (Biotek, Winooski, VT) using an excitation of 485/13 nm, emission of 528/17 nm, and a gain of 50, with measurements being made from the bottom of the well plate.

### 2.5 | Autosampling

Dilutions of HeLa digest stock solutions were prepared at 0.25, 0.5, 2.5, 5, 10, and 20 ng/μL in solvent A. Dilutions were loaded in 10μL aliquots into 384-well plates maintained at 4° C using an OT-2 tempdeck module. Sample injections were prepared by aspirating either 500 or 1700 nL of sample followed by 3500 or 3000 nL of solvent A for underfilling or overfilling of the sample loop, respectively. Underfilling was used to inject samples from the 0.5, 5, and 20 ng/μL dilutions. Overfilling of the sample loop was used to inject samples from the 0.25, 2.5, and 10 ng/μL dilutions. In all cases, the sample loop was flushed between injections with 25 μL of solvent A.

### 2.6 | LC Methods

Separations were performed with a flow rate of 200 nL/min. The UltiMate 3000 pump was programmed to wait for a signal from our software to indicate that sample loading was finished. Upon receiving the signal, the pump first increased solvent B from its initial 1%–5% in 1 min, then ran an elution gradient of 5%–25% B over 20 min. Following the elution gradient, solvent B was ramped from 25 to 45% in 2 min, then from 45% to 75% in 1 min. The pump was held at 75% B for 4 min before ramping back down to 25% in 1 min and being held there for another minute. The pump was then ramped back to 75% B in 1 min, held at 75% for 3 min, and then ramped down to 1% in 1 min, where it remained for the last 15 min of the protocol.

### 2.7 | MS Methods

An Orbitrap Exploris 480 mass spectrometer (Thermo Scientific) was used for acquisition of LC-MS data. Electrospray was performed using a Nanospray Flex Ion Source with a potential of 2200 V. The ion transfer tube was set to 250°C. MS1 scans were performed at a resolution of 120K with a scan range of 375–1575 Th, the RF lens at 50%, a normalized ACG target of 300%, and a maximum injection time of 22 ms. To trigger MS/MS in data-dependent acquisition mode, the precursor intensity threshold was set to 5.0e3, charge state was set at 2–6, and dynamic exclusion window was set at 60 s. For experiments of 2.5- or 10-ng injections, MS/MS cycle time was set to 1.5 s, the isolation window was set to 10 Th, HCD collision energy was 30%, and the resolution was set to 45 000. Experiments for 250 pg injections used the same settings except for an MS/MS resolution of 90 000.

### 2.8 | LC-MS Data Analysis

The number of proteins/peptides identified was first processed through MSConnect after each MS run. MSConnect is a web-based data management platform that links MS files to various vendors’ data search software for processing and provides real-time protein/peptide identification results [47]. In this study, FragPipe v22.0, incorporating MSFragger v4.1, IonQuant v1.10.27, and diaTracer v1.1.5, was used for DDA+ data processing. Each MS file was processed individually, without match between runs or intensity normalization across runs. The human FASTA database was downloaded from UniProt on July 7, 2020, and decoy sequences along with contaminants were added using FragPipe. Precursor mass tolerance was set to 20 ppm, with strict trypsin enzymatic digestion. Peptide length was restricted to 7–50 amino acids within an *m*/*z* range of 500–5000, and a 0.4-min retention time window was applied. Cysteine carbamidomethylation (+57.0215 Da) was set as a fixed modification, while methionine oxidation (+15.9949 Da) and N-terminal acetylation (+42.0106 Da) were considered variable modifications, allowing a maximum of three variable modifications per peptide. Quantification was performed at the MS1 level. A second analysis with match between runs (MBR) was performed with retention time tolerance of 0.25 min and a *m*/*z* tolerance of 5 ppm.

## 3 | Results and Discussion

### 3.1 | Developing Open-Source Software for Custom LC Systems

**FIGURE 4 |:**
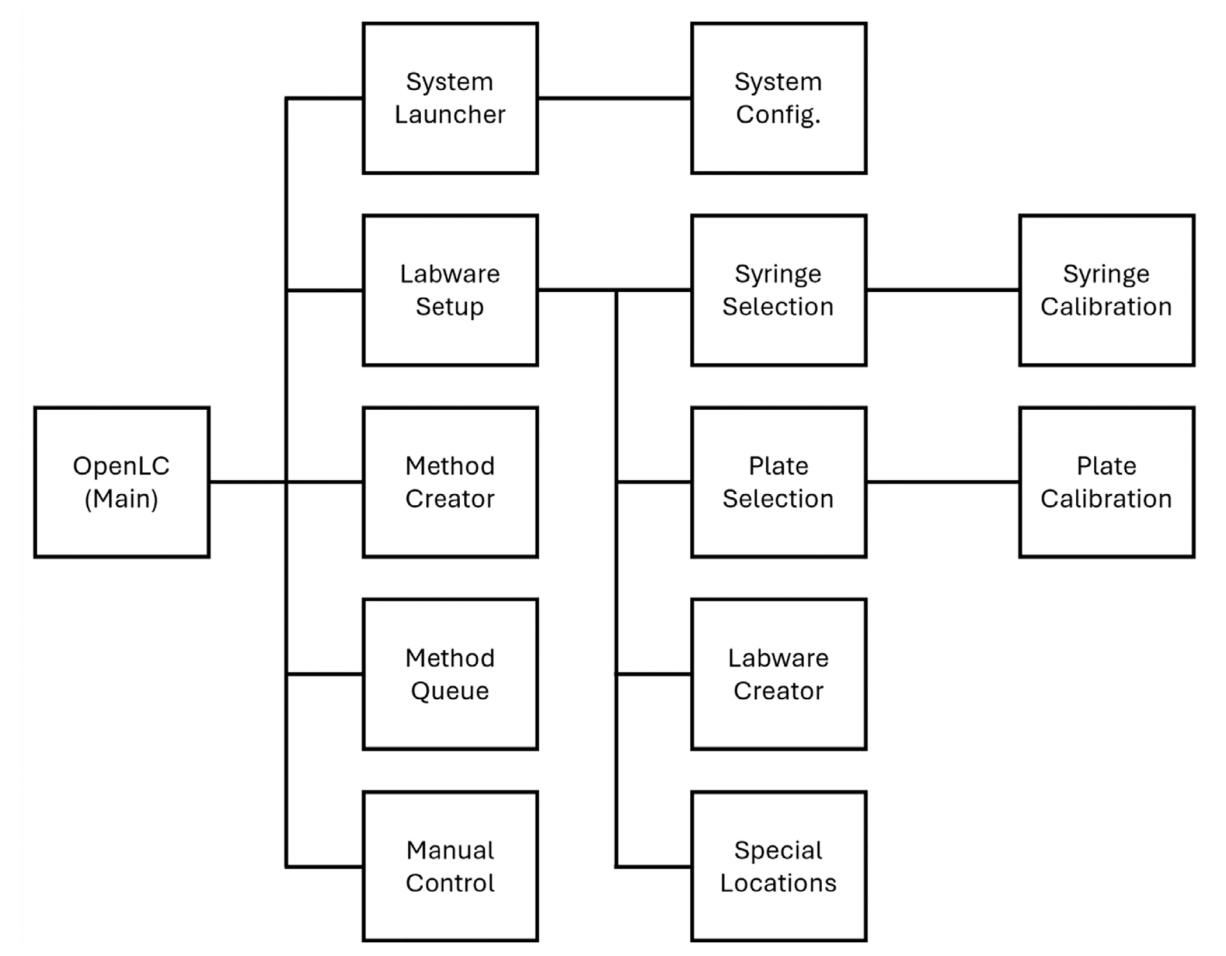
A system diagram of OpenLC software’s user interface. This software enables custom system configuration, labware declaration and calibration, method editing and execution, and manual system control.

While Opentrons systems are open source and modifiable, both their hardware and their API have been developed primarily for automation of large-scale pipetting workflows. We were able to perform simple nanoscale liquid handling routines using the original OT-2 software and electronic components, with the only physical changes being to one of the detachable pipettes. To do this, we converted target volumes first into linear distance, then linear distance into corresponding microliter volumes for an unmodified pipette. We developed a conversion table for this, based on the piecewise functions the Opentrons software uses for its pipette operations. For more advanced applications involving integration of external components, such as LC autosampling, we determined that alternative software was needed. For this, we developed a Python software package (Figure 4), termed OpenLC, for flexible configuration of homebuilt LC systems. OpenLC can be used to assemble systems with modular components including Zaber linear actuators, relays, generic inputs and outputs, and VICI valve actuators for 2-position and selector valves. By incorporating the OT-2 motor drivers into our OpenLC software, we could coordinate the OT-2 XYZ and pipette motors, and an OT-2 Tempdeck module, with these other components used in our home-built systems. Though initial setup of such systems will benefit from familiarity with the various components, OpenLC provides a user interface to increase accessibility of configuration and operation. After initializing a configuration, users can define and calibrate labware, draft method files, queue up execution of method files, and manually control system components as needed.

### 3.2 | Modifying an OT-2 for Sub-Microliter Liquid Handling

In its native state, the OT-2 system is controlled by a raspberry pi, configured by Opentrons to run Python scripts using predefined functions to provide the system’s motor controllers with step-by-step instructions. A small circuit board attached to the raspberry pi (included as part of the OT-2) has a “debug” switch that allows external computers to bypass the raspberry pi and interface directly with the motor controllers. We used jumper wires to connect the debug pins to an FTDI chip, which was in turn connected to the operating computer via USB cable. A 3D-printed extension was affixed to this switch for convenience. Toggling of the switch enables easy conversion between operating the OT-2 with its native software and controlling it with OpenLC.

Physically adapting the OT-2 builds directly upon our previously reported work to adapt an Opentrons OT-1 for nanoliter liquid handling [41]. The OT-1 made use of a stepper motor to operate mounted hand-held pipettes. A key difference between their first- and second-generation platforms was the introduction of Opentrons electronic pipettes, which incorporated the stepper motor-based actuation mechanism directly into the pipette. Because these pipettes are easily exchanged, we were able to add syringe control to the OT-2 while preserving the native pipetting capabilities of the system. We selected a Gen2 single-channel pipette for syringe control (Figure 2A,B) and adapted it as follows.

The pipette’s cover plate, piston, and shaft components were removed, leaving the frame, stepper motor components, and the plastic attachment for the lead screw of the motor. A 3D-printed piece designed for operating a syringe plunger was attached to the drive shaft of the pipette. A separate piece designed for mounting the syringe was affixed to the bottom of the pipette frame. An arm extends from one side of the syringe mount for the purpose of attaching a camera to the syringe mount. A threaded rod was affixed to the arm with nuts and washers. A 3D-printed piece was designed to mount a digital microscope camera to the threaded rod to allow for ease in adjusting the camera view.

To add foil-punching functionality, we modified a second pipette (Figure 2C), selecting a Gen1 model because the lower quality internal motor was not a factor. The pipette cover plate, piston, and shaft components were removed and replaced with an attachable 3D-printed extension that was designed for puncturing small holes in the sealing foil over individual wells.

### 3.3 | Configuring the Opentrons OT-2 for Autosampling and LC

Two 3D-printed actuator mounts were attached to screw guides on the back of the z-axis framework of the OT-2 (Figure 1B). A gastight syringe was installed in the modified pipette as described in Section 2 (Figure 2B). VICI actuators were affixed to the mounts and fitted with an eight-position selector valve (upper) and a six-port two-position valve (lower). The valves were configured for autosampling applications as shown in Figure 3. The syringe buffer line was connected to the center port of an eight-position selector valve, which enabled flushing and refilling of the syringe as needed. The other port positions of the selector valve were chosen arbitrarily as follows. Port 1 of the selector valve was connected to a waste line. Port 3 was connected to a vial of solvent A. Port 6 of the selector valve was connected to port 1 of the two-position valve. The remaining ports of the selector valve were unused for this configuration. A capillary connecting ports 2 and 5 of the two-position valve served as the sample injection loop. A capillary sampling needle was connected to port 6. The sampling end of the needle was affixed to the syringe mount to allow three-axis automated positioning. Port 3 of the two-position valve was connected to an HPLC pump. Finally, port 4 was connected to the separation column.

The control pins of the valve actuators were connected to general-purpose input/output (GPIO) pins of an ESP32-WROOM-32 module (Espressif Systems, Shanghai). The module was previously flashed using the Arduino IDE software with a script, which enables GPIO configuration based on serial communication from our software. This module is also used to send and receive signals to and from external devices. Our system was configured to trigger the LC gradient method through a relay module and receive end-of-method signals from the pump through a GPIO pin.

### 3.4 | Characterization of Sample Handling

**FIGURE 5 |:**
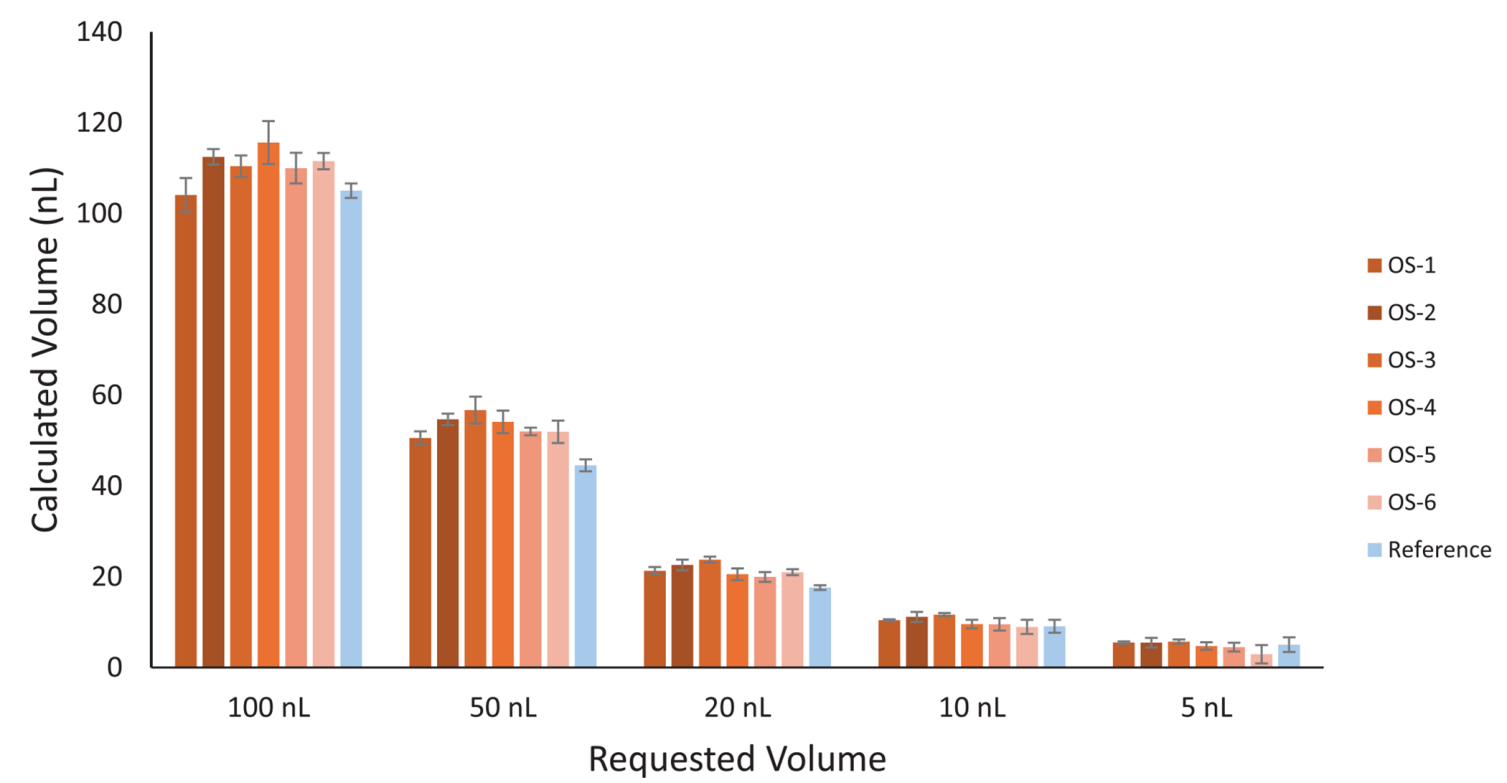
Characterization of the accuracy and reproducibility of dispensing sub-microliter volumes using the OpenSampler (a modified Opentrons OT-2). The OpenSampler system was programmed to dispense fluorescein aliquots into buffer-filled wells. Fluorescence was measured against dilute, hand-pipetted standards to determine aliquoted volumes. The comparison was performed six times using the OpenSampler software (OS-1 through -6) and once using the original OT-2 software (Reference), with error bars representing CVs for each.

Liquid handling in the microliter range is commonly characterized using gravimetric analysis. This technique becomes difficult to employ for sub-microliter volumes where such changes in mass are prone to evaporation-related errors. An alternative to gravimetric analysis is fluorimetry, which relies on the concentration-dependent intensity of fluorescent materials. Fluorescence measurements are also dependent on pathway length, making it error prone when directly measuring such small volumes using typical fluorimetry equipment. The advantage of using fluorimetry is that it is a highly sensitive technique. We can indirectly measure sub-microliter aliquots of fluorescein by dispensing them directly into large aliquots of solvent. The relatively large solvent volumes provide sufficient path lengths for analysis and reduce the impact of evaporation-related errors. We programmed our OpenSampler to dispense 5, 10, 20, 50, and 100 nL aliquots ( *n* = 5 for each volume) of fluorescein standards into wells prefilled with 20 μL of solvent. We measured the fluorescence of the diluted mixtures alongside a series of standards. Grubb’s tests were performed for each volume set within each batch and for the standards. This resulted in a few eliminations in both the mixtures and the standards, which we attributed to errors made while hand pipetting. We then calculated the dispensed volumes based on the known original concentrations to determine the final concentrations of the mixtures (Figure 5). All tested volumes had absolute errors of less than 15%, with CVs < 7% for dispensed volumes of 20 nL or more for each batch. Individual batches were each measured on different days. Volumes < 100 nL are typically used for reagent addition in individual sample preparation steps, and an absolute error in volume will alter the amount of, for example, protease introduced. However, proteomic sample preparation is quite tolerant to a range emzyme to substrate ratios, so the impact on sample preparation is expected to be minimal. For LC sample injections, we recommend injection volumes > 100 nL, where volumetric accuracy and precision substantially improve.

### 3.5 | LC-MS Analysis Using the OpenSampler System

**FIGURE 6 |:**
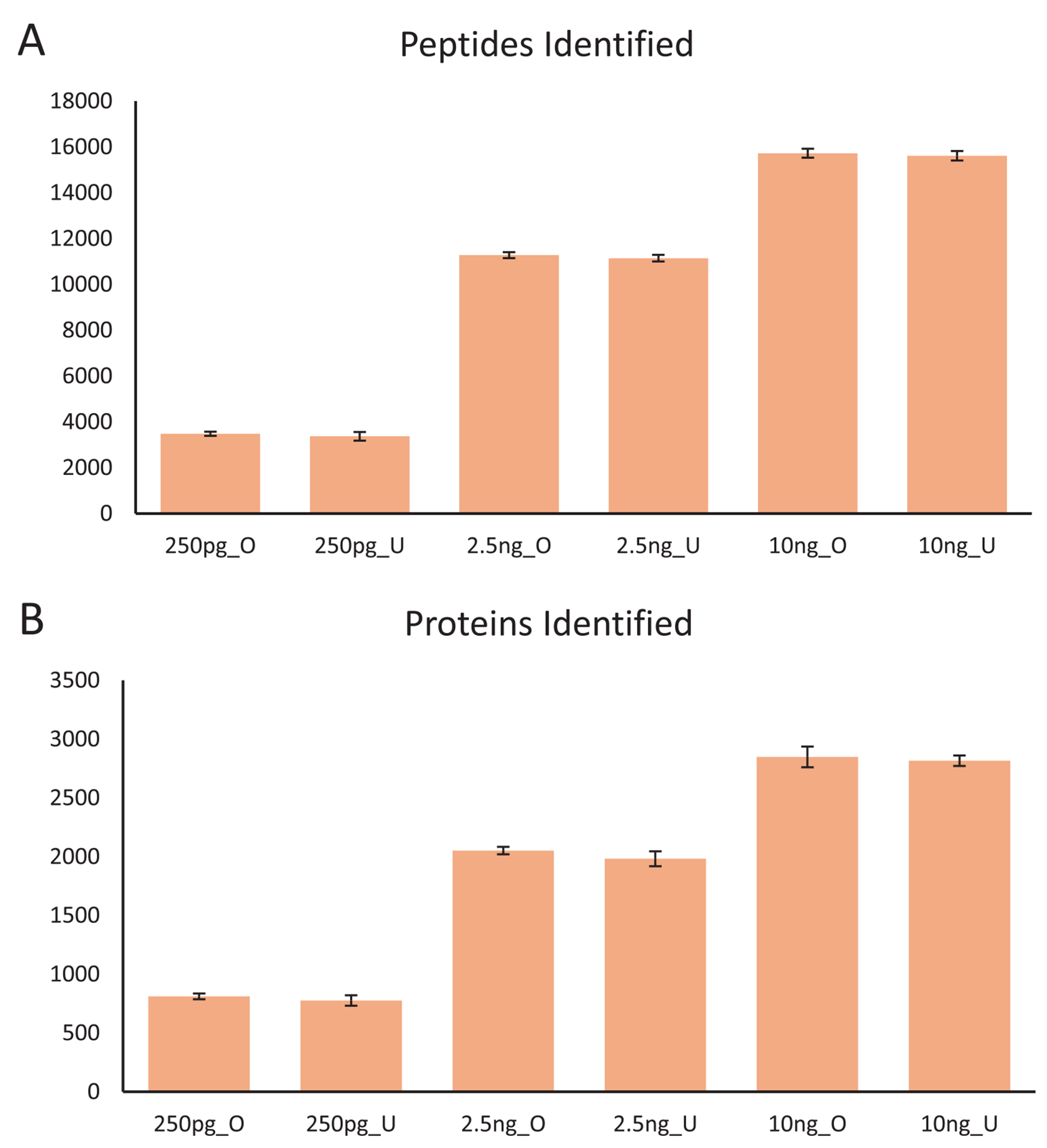
Average number of peptides (A) and proteins (B) identified by WWA analysis of injections of QC HeLa digest ( *n* = 3, error bars are ±1 standard deviation). Digest standards were sampled either by overfilling (“_O”) or underfilling (“_U”) the sample loop to target injections of 0.25, 2.5, or 10 ng of digest.

LCMS analyses of HeLa digests were performed to both demonstrate and characterize the autosampling capabilities of the OpenSampler system. Both underfilling and overfilling the sample loop (aspirating less or more sample than the sample loop’s volume capacity) were evaluated for this purpose. Underfilling is ideal for sample-limited studies but is more susceptible to errors from volume-dependent reproducibility. Overfilling relies on the sample loop volume to ensure reproducible injections. Six concentrations were selected to target underfilling or overfilling a sample loop with 0.25, 2.5, and 10 ng of protein digest, approximating sample input corresponding to 1, 10, or 40 mammalian cells. An aliquot of each digest concentration was analyzed in ascending concentration in each of two batches, with two blank injections between each batch. Three replicate injections were analyzed from each aliquot. To limit evaporation, the foil seal for each aliquot was punctured immediately before the first injection, with replicate analyses following immediately. The overfilled injections resulted in the identification of an average of 811,2052, and 2848 protein groups for 0.25, 2.5, and 10 ng injections, respectively (Figure 6). The underfilled injections resulted in the identification of an average of 776, 1982, and 2816 protein groups, respectively. Identified proteins and peptides were evaluated for statistical differences between the triplicate groups of each condition and for differences between the two sampling strategies at each protein input level, with no statistically significant differences being observed in either case.

## 4 | Conclusions

We developed OpenLC, a software for customizable nanoLC systems to enable the development of custom liquid handling and autosampling workflows. This software significantly reduces the technical expertise required to assemble and operate custom systems. We demonstrated the applicability and flexibility of this software by adapting a popular low-cost pipetting robot for sample preparation and autosampling for low-input proteomics. We evaluated the sub-microliter liquid handling of our OpenSampler system and found the reproducibility suitable for handling volumes typical of trace proteomics workflows. We then demonstrated the autosampling capabilities of our system by injecting as little as 250 pg of protein digest, which is comparable to single-cell levels. Together, these initial results show the feasibility of using this system in a wide variety of LC-MS studies. This work could be furthered by studies to demonstrate transferability to additional OT-2 systems and evaluation of sample stability over larger set.

A key aspect of our software is the ability to develop systems capable of accomplishing tasks outside the scope of commercially available systems or to combine capabilities into a single system. The nano volume valves used in this system can be easily reconfigured or replaced with other valves to enable a variety of LC strategies, such as trap-and-elute injections. The low cost and flexible nature of the OpenSampler system makes it a viable option for resource-constrained labs aiming to develop custom liquid handling and LC instrumentation.

## Data Availability

The OpenLC software and OpenSampler stl files are available on github: https://github.com/RTKlab-BYU/OpenLC and https://github.com/RTKlab-BYU/OpenSampler/tree/main
